# Cognitive and Neural Mechanisms of Behavior Therapy for Tics: A Perception–Action Integration Approach

**DOI:** 10.3390/biomedicines11061550

**Published:** 2023-05-26

**Authors:** Julia Friedrich, Tina Rawish, Annet Bluschke, Christian Frings, Christian Beste, Alexander Münchau

**Affiliations:** 1 Institute of Systems Motor Science, Center for Brain, Behavior and Metabolism (CBBM), University of Lübeck, 23562 Lübeck, Germany; julia.friedrich@uni-luebeck.de (J.F.); tina.rawish@uni-luebeck.de (T.R.); 2 Cognitive Neurophysiology, Department of Child and Adolescent Psychiatry, Faculty of Medicine, Technische Universität Dresden, 01307 Dresden, Germany; annet.bluschke@ukdd.de (A.B.); christian.beste@ukdd.de (C.B.); 3 Department of Cognitive Psychology, Faculty of Psychology, University of Trier, 54296 Trier, Germany; chfrings@uni-trier.de; 4 University Neuropsychology Centre, Faculty of Medicine, Technische Universität Dresden, 01307 Dresden, Germany; 5 Cognitive Psychology, Faculty of Psychology, Shandong Normal University, Jinan 250014, China

**Keywords:** Gilles de la Tourette syndrome, tics, CBIT, HRT, ERP, theory of event coding, TEC, binding and retrieval in action control, BRAC

## Abstract

European clinical guidelines recommend the use of Exposure and Response Prevention (ERP) and Comprehensive Behavioral Intervention for Tics (CBIT) as first-line treatments for tic disorders. Although ongoing efforts in research are being made to understand the mechanisms underlying these behavioral approaches, as of yet, the neurophysiological mechanisms behind behavioral interventions are poorly understood. However, this is essential to tailor interventions to individual patients in order to increase compliance and efficacy. The Theory of Event Coding (TEC) and its derivative BRAC (Binding and Retrieval in Action Control) provide a theoretical framework to investigate cognitive and neural processes in the context of tic disorders. In this context, tics are conceptualized as a phenomenon of enhanced perception–action binding, with premonitory urges constituting the perceptual and the motor or vocal expression constituting the action part of an event file. Based on this, CBIT is assumed to strongly affect stimulus–response binding in the context of response selection, whereas the effects of ERP presumably unfold during stimulus–response binding in the response inhibition context. Further studies are needed to clarify the neurophysiological processes underlying behavioral interventions to enable the individualization and further development of therapeutic approaches for tic disorders.

## 1. Introduction

Gilles de la Tourette syndrome (GTS) constitutes a multifaceted neurodevelopmental disorder defined by several motor tics and at least one vocal tic persisting for more than one year [1]. Based on its cardinal diagnostic features, which are increasingly debated [2,3], GTS is typically categorized as a movement disorder, although most tics are associated with premonitory urges [4,5] and other sensory phenomena [6,7]. Moreover, tics have been related to increased behavioral habit formation tendencies [8]. Therefore, it has previously been suggested to conceptualize GTS as a disorder of perception–action integration [4].

Tics can be temporary and frequently go partly unnoticed in childhood as they often subside without treatment; however, in some individuals, tics can cause disability [9]. In the past few decades, numerous treatment approaches for tic disorders have been developed and studied, including psychoeducational and pharmacological approaches, with a particular focus on behavioral interventions [10]. European clinical guidelines recommend the use of behavior therapy, namely Exposure and Response Prevention (ERP) and Comprehensive Behavioral Intervention for Tics (CBIT), as first-line treatment for tic disorders in children and adults [11]. There is ample evidence to suggest that Habit Reversal Training (HRT) and, more specifically, its extended version, CBIT, is an effective intervention for tic disorders in children [12,13] and adults [14]. ERP is also recommended as a first-line behavioral treatment for tic disorders in Canadian, European, and American guidelines [11,15,16,17], although there is less certainty regarding the effectiveness of this treatment due to there being less empirical evidence regarding this treatment compared to HRT/CBIT [11]. Ongoing efforts in research are being made to understand the mechanisms underlying these behavioral approaches. This is essential not only to explore how a therapeutic intervention contributes to an improvement in general but also to potentially tailor interventions individually to a given patient to increase effectiveness and compliance. Furthermore, it provides the opportunity to offer patients the intervention that is most likely to be effective based on an evidence-based predictive model (e.g., using biomarkers) [18]. In addition, a detailed understanding of the underlying mechanisms of an intervention can create a basis to further develop and refine this method and also provide further insights into GTS pathophysiology.

As of yet, the cognitive and neural mechanisms behind behavioral interventions are poorly understood [19]. A change in the conceptualization of tic disorders from a classical movement disorder towards a disorder of perception–action integration not only opens up new possibilities for the investigation of the cognitive and neural processes underlying tic disorders but also for the investigation of the underlying effects of tic-specific behavior therapies. In the following sections, we argue that an overarching theoretical framework integrating action and perception, i.e., the Theory of Event Coding (TEC) and its derivative, the BRAC (Binding and Retrieval in Action Control) concept, may be used for a better understanding of tic disorders because these treatments provide a new perspective on disorder- and treatment-specific behavioral and neural processes. In order to do so, a detailed summary of the behavioral treatment approaches for GTS and other tic disorders and their effectiveness will be followed by an overview of the theoretical frameworks of TEC/BRAC and a subsequent presentation of the conceptual link between these aspects.

## 2. Behavioral Therapy Approaches in the Treatment of GTS

### 2.1. Comprehensive Behavioral Intervention for Tics (CBIT)/Habit Reversal Training (HRT)

HRT and CBIT are well established in the behavioral treatment of tic disorders [15]. CBIT is an extension of HRT by another core component (function-based interventions) and comprises several additional elements such as relaxation training and the introduction of an age-appropriate reward contingency system as a therapeutic element to maintain motivation during the course of the treatment.

According to Woods et al. [20], CBIT treatment starts by focusing on disorder- and treatment-specific psychoeducation as well as a comprehensive and detailed description of all the tics that are currently present. In subsequent sessions, each tic is addressed individually, with both core components (HRT and function-based interventions) forming an integral part of each session.

Firstly, in order to develop function-based interventions, triggering and reinforcing factors for each specific tic are analyzed (i.e., function-based assessment). Subsequently, individual function-based interventions that aim to reduce these influencing factors or their consequences as well as reduce the tic frequency and/or severity and, consequently, the distress caused by tics are developed. A wide variety of behavioral interventions such as cognitive restructuring or practicing social skills can be applied in this step; however, the most important part is to teach patients to make use of the competing response developed in HRT as a coping strategy [20].

Function-based assessment and the development of function-based interventions are followed by HRT. In the first step, awareness training is conducted to improve tic detection and increase awareness of the preceding urge. For this purpose, patients provide a detailed description of the tic and the premonitory urge, followed by a training focusing on the perception/detection and indication of occurring tics and urges. Awareness training is an important prerequisite to change the automated response (i.e., the tic) that follows the urge. During awareness training, the link between triggering factors and tics is particularly highlighted [12,14]. However, it has to be pointed out that the association between urges and tics may not be as straightforward as is commonly assumed. A study by Langelage et al. [21] revealed variable associations between tics and urges in children at the individual level, with a large proportion of participants showing no and some even a negative association between urges and tics. In adult patients, Schubert et al. [22] found a positive association between urges and tics only at the group level, whereas relations between urges and tics were variable at individual levels. However, awareness training and HRT can also be applied even if no urge is perceived [23]. Subsequently, competing response training teaches patients to engage in tic-incompatible competing behavior instead of performing the tic [20]. The incompatible response should be applied whenever the urge or the tic occurs. It should then be maintained for at least one minute or until the premonitory urge has markedly reduced. However, even in patients who do not experience premonitory urges, an incompatible response can successfully be established [20,23]. Patients are also required to complete homework to foster learning effects and train their ability to transfer the learned skills into daily life settings.

HRT, as a core component of CBIT, is effective in reducing GTS symptoms [24]; however, the exact cognitive and neural mechanisms underlying its effectiveness are still not clear. There are hypotheses suggesting that activity changes in basal ganglia circuits relevant in GTS [25,26,27] or habituation to premonitory urges mediate intervention effects [20]. However, those explanations are not specific, and there is a lack of empirical evidence on this topic. Importantly, only with a better mechanistic understanding of the underlying cognitive and neural mechanisms, reasonable attempts can be made to increase the effectiveness of behavioral treatments in GTS.

### 2.2. Exposure and Response Prevention (ERP)

**Figure 1:**
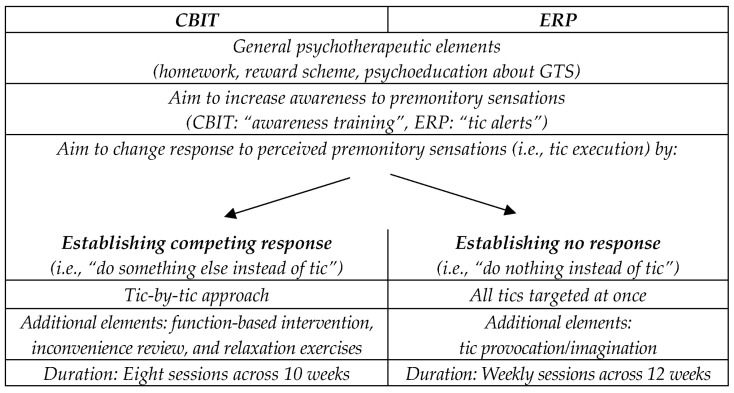
Comparison between CBIT and ERP with respect to general psychotherapeutic elements, aims, and duration of the interventions.

RP assumes that tics can be conceptualized using the framework of negative reinforcement. In this context, tics are conditioned responses to (for example) sensory stimuli and are performed to eliminate the unpleasant sensation (i.e., urges) [23,28,29,30]. Verdellen et al. [31] presented a manual for the treatment of children and adolescents with ERP. Unlike HRT/CBIT, ERP addresses all tics simultaneously. During initial sessions of ERP, patients train to suppress the execution of all their tics (i.e., response prevention). Subsequently, they are instructed to concentrate on premonitory urges (i.e., exposure) to habituate to the unpleasant urges [32,33]. This becomes increasingly difficult, gradually incorporating triggering factors (e.g., tic-triggering activities, imagination) into the exercises in order to provoke tics. Additionally, the therapist regularly collects information on the strength of the urges and redirects the patient’s focus to them [11]. In addition, an age-appropriate reward scheme can be used, and patients are also required to complete homework to practice tic suppression at home and integrate strategies into everyday life. The training can be supplemented by other elements, such as relaxation exercises. The assumption of habituation as an underlying mechanism is not undisputed [24,34]. Figure 1 provides an overview of CBIT and ERP and summarizes general procedures, similarities, and differences.

## 3. Current Evidence on the Effectiveness of CBIT/HRT and ERP for the Treatment of Tic Disorders

Since the early 2000s, several randomized controlled trials assessing the effectiveness of HRT, particularly in contrast to supportive psychotherapy (SP) or waitlist control conditions, have been published. Piacentini and colleagues (2010) examined 126 children and adolescents before and after treatment (CBIT or SP plus psychoeducation). Compared to the SP group, children and adolescents treated with CBIT showed a significant reduction in total tic score [12], which was measured by using the Yale Global Tic Severity Scale (YGTSS) [35]. Several other studies investigated the effectiveness of HRT or CBIT compared to SP in adult patients, likewise using YGTSS symptom severity assessment as a primary outcome measure. Again, there was a significantly larger reduction in tic severity in the group of patients receiving CBIT or HRT compared to the SP group [14,36,37]. In a meta-analysis of eight randomized-controlled trials, McGuire and colleagues (2014) found medium to high effect sizes for the effectiveness of HRT compared to waiting list or other control conditions (mostly SP). It was shown that the effect size was age-dependent, with older subjects tending to benefit more from the therapy than younger participants. This could be interpreted as an increased readiness of older subjects to integrate strategies learned in therapy into everyday life or be attributed to a superior perception of premonitory urges among older subjects, which is a core component of HRT [38]. This is in line with the above-mentioned findings of Langelage et al. [21], showing a tendency towards weaker urge-tic association in children and adolescents compared to adults. However, only two studies with adolescent participants were included in the meta-analysis, meaning that the informative value of this finding is limited [38]. Further studies published since 2014 in child and adolescent samples have demonstrated the effectiveness of HRT and CBIT in contrast to waiting list conditions or control interventions (e.g., psychoeducation) in this age group [39,40]. However, with regard to the study by Rizzo and colleagues, it must be taken into account that the behavior therapy group consisted of patients who received either HRT or ERP with no separate evaluation of the effectiveness of each intervention for the two groups [39]. In a more recent meta-analysis, which also assessed the effectiveness of HRT, the analysis of 10 studies (586 patients) showed a small to medium effect size for HRT depending on the control condition applied [41]. However, it should be noted that, on the basis of the studies described, no profound statements can be made regarding the long-term effects of the interventions due to missing or inconclusive follow-up evaluations. In fact, Dutta and Cavanna [42] identified methodological limitations upon conducting follow-up measurements. These limitations consist of a significant drop-out of participants at the time of follow-up [12,36], the lack of validity of the follow-up assessment due to interventions that have been conducted in the meantime (cross-over design) [30], and the exclusive inclusion of treatment responders in the follow-up assessment [12]. In one study, a follow-up interval of 10 months was chosen [37]. It was demonstrated that the YGTSS total tic score of the HRT group was no longer significantly different from the SP group at this point. This was due to a slight increase in the YGTSS score in the HRT group but was mainly attributed to a decrease in the SP group [37]. Further studies are urgently needed to investigate the stability of treatment effects.

As mentioned above, there is far less empirical evidence for the effectiveness of ERP in comparison to CBIT/HRT in the treatment of tic disorders [11]. However, it has been shown in a randomized controlled trial that HRT (as one core component of CBIT) and ERP achieve similar results with regard to reducing tic severity and frequency [30]. An open study by Andrén et al. [43] examined the effectiveness of behavioral therapy (predominantly psychoeducation and ERP, as well as psychoeducation and HRT and “other behavioral therapy”, such as psychoeducation and a combination of ERP and HRT) in a naturalistic setting. Approximately 57% of treated patients were classified as treatment responders across all treatment groups using the Clinical Global Impression-Improvement scale (CGI-I) [44] at post treatment. Furthermore, there was a significant decrease in tic severity and tic-related impairment using the YGTSS as an outcome measure. However, it should be noted that the results have to be interpreted with caution especially due to the lack of a control group and randomization [43].

Despite behavioral therapy recommendations, it is hard to find a therapist who is qualified to perform this treatment [45]. One way to improve this situation is to provide treatment delivered via videoconference so that restrictions due to a lack of mobility, large distance, or other logistic obstacles no longer prevent patients from receiving treatment. It has been shown that tic severity is reduced following CBIT regardless of whether the treatment is delivered in person or online [46]. This underscores that the essential mechanisms of action are addressed independently of the setting. However, the lack of trained therapists is not resolved by online therapy. Fortunately, it has been shown that CBIT-based internet-delivered training programs [40,47] or combined CBIT- and ERP-based approaches [48] reduced impairment associated with tics and led to high patient satisfaction.

## 4. The Relevance of Neurophysiological Markers

In order to gain insights into the mechanisms underlying treatment effects, a few studies have assessed the impact of behavior therapy for tics on neurophysiological processes. However, the question of the mechanisms of action underlying effects of (recommended) behavior therapies on tic disorders at the neurophysiological level is far from fully clarified. Answering this question could be beneficial for the specification and individualization of therapeutic approaches. One previous study investigated neural correlates of behavior therapy by comparing neural activation in a response inhibition (Visuospatial Priming) task before and after CBIT using functional magnetic resonance imaging (fMRI) in eight patients with GTS and eight healthy controls [49]. They found increased activation in the putamen in patients with GTS before treatment compared to healthy controls and decreased putamen activation after treatment indicating stronger changes in putamen activation. Furthermore, they found a negative correlation between changes in tic severity, as indicated by the YGTSS total tic score, and a change in inferior frontal gyrus activation from before to after intervention. It was concluded that CBIT may contribute to the normalization of deviant basal ganglia activation involved in tics. However, the authors emphasized that these findings are rather preliminary due to the small sample size and the lack of an active control group [49].

Due to its excellent temporal resolution, electroencephalography (EEG) is a valuable method for mapping brain dynamics. However, there seems to be a gap in research concerning studies on the neurophysiological correlates of behavior therapy. This could provide a basis to extract markers associated with treatment effects. A previous study involving children and adolescents with GTS found that the subjects’ neurocognitive status, assessed using different neuropsychological tests (e.g., of inhibitory functions and habit learning) before CBIT, was not significantly changed after treatment and did not predict treatment outcome [50]. Electrophysiological processes may be more suitable to provide reliable predictors of tic symptom reduction and may allow the individual allocation of therapy to different patients based on markers extracted before treatment [18]. One study [18] investigated electrophysiological processes in a stimulus–response compatibility task underlying cognitive-behavioral therapy (CBT), or more precisely, cognitive-psychophysiological therapy constituting an alternative cognitive-behavioral approach including biofeedback and psychophysiological exercises to differentiate muscle tension levels [51,52]. In contrast to the implementation of competing responses or exposure to premonitory urges, as is the case with CBIT and ERP, respectively, cognitive-psychophysiological therapy was designed to modify physiological processes underlying the tic rather than the tic itself [52]. Whereas the classical compatibility effect did not differ between patients and healthy controls on a behavioral (reaction time) level, they found differences between adult patients with tic disorders and healthy control participants with respect to stimulus- and response-locked lateralized readiness potential (sLRP and rLRP, respectively) before CBT. In healthy controls, neurophysiological processes did not change significantly, whereas, among patients, delayed sLRP onset was accelerated, and the larger rLRP amplitude was reduced in incompatible trials after CBT. Behavioral performance as well as the more frontal than central distribution of the NoGo-P3 (NoGo-Anteriorization) in patients was not affected by the intervention [18]. The variance in tic reduction was measured to be around 43% after the intervention, which could be explained using a multiple linear regression analysis encompassing the N2 and the sLRP in the incompatible condition. A larger N2 amplitude in incompatible trials before cognitive-behavioral treatment was associated with better treatment response. It has to be noted that, despite encompassing some elements of recommended behavior therapies [51], the intervention used in the latter study is currently not part of behavior therapy recommendations in European guidelines due to a lack of randomized controlled trial data on treatment effects [11]. Although this review focuses on the recommended two first-line behavioral treatments, CBIT and ERP, the findings of the above-mentioned study investigating an alternative cognitive-behavioral approach using a stimulus–response compatibility task indicate that the way in which incompatibility between stimuli and responses are processed seems to play a role in cognitive-behavioral therapy [18]. Importantly, behavioral performance was not linked to treatment outcome, underlining the necessity to include markers of neurophysiological activity underlying stimulus–response interference tasks [18].

One study investigated children with GTS in a randomized controlled trial using a Go/NoGo task [53]. The authors examined whether CBIT modulates EEG biomarkers, i.e., alpha coherence, frontal midline theta, and event-related potentials (N2 and P3), and whether these markers are potential predictors of treatment response. Aside from providing further evidence that CBIT effectively reduced tics in children with GTS, it was reported that CBIT did not affect behavioral performance in this cognitive control task. Additionally, EEG markers were not affected by CBIT and did not predict treatment outcome.

In summary, further research is needed to explore the neural mechanisms underlying treatment effects using theoretical explanatory models as a basis. In light of recent findings, the Theory of Event Coding (TEC) and its derivative BRAC (Binding and Retrieval in Action Control) seem to be suitable for the investigation of neural and cognitive processes, especially in the context of tic disorders. Conceptualizing GTS and chronic tic disorders as disorders of perception–action integration opens up new possibilities for investigating and understanding the underlying neurophysiological processes [4,54,55,56,57].

## 5. Theory of Event Coding (TEC) and Binding and Retrieval in Action Control (BRAC)

The TEC is an overarching framework that integrates action and perception by specifying how stimulus features are associated with response features, i.e., motor processes [58]. Within the TEC framework, this is referred to as “binding” [58,59], i.e., the integration of features defining a stimulus (e.g., color or location) and features characterizing an action/response (e.g., response hand, movement distance). Whereas stimulus features are stored in “object files”, action features are combined in “action files”, and, importantly, perception and action features are jointly stored and processed in “event files”. In an event file, stimulus and response features are bound [60,61,62]. The event file represents a network of neural representations of stimulus and response features and their connections or bindings [63]. Because of this network structure, (re-)encountering one stimulus or response feature is enough to (re-)activate to whole event file [64], which then affects response selection. If an event file is established but a previously encountered stimulus now requires a different response (or another stimulus requires the same response as performed previously), the “old” stimulus–response association is not valid anymore. Therefore, the event file has to be reconfigured, resulting in prolonged response times and higher error rates (in TEC terms, this is referred to as “partial repetition costs”) [60,62]. If all or no stimulus and response features are repeated, performance (i.e., accuracy or reaction time) is superior compared to trials involving the partial repetition of stimulus or response features.

BRAC represents an extension of TEC, aiming at specifying processes of action planning and execution (i.e., how event files are dynamically managed) but also pursuing the goal of integrating different research approaches in the field of action control into a perception–action perspective [65,66]. This conceptual framework distinguishes binding and retrieval processes as the most important aspects governing how event files are managed. According to BRAC, the partial repetition costs described in the TEC arising during the reconfiguration of event files can, on the one hand, be caused by effects of unbinding and rebinding. On the other hand, it can be argued that partial repetition costs arise when an existing event file is retrieved. This means that the magnitude of partial repetition costs (i.e., the behavioral binding effect) cannot indicate whether these costs are due to binding or retrieval processes, or both. A measured binding effect always comprises a binding proper plus retrieval. Furthermore, top-down (e.g., attentional weighting) [67] or bottom-up processes (e.g., perceptual grouping of distractors) [68] can influence binding and retrieval independently. The distinction between binding and retrieval motivates experimental manipulations that allow more precise insights and predictions of behavioral and neurophysiological processes so that treatment approaches could potentially be tailored to altered cognitive processes [65].

## 6. GTS as a Disorder of Perception–Action Integration

Linking the TEC and BRAC framework to GTS, the assumption that actions are based on and are prerequisites for perception is of central importance [62,63,69]. On this basis, tics as a whole can be conceptualized as a phenomenon of perception–action integration, i.e., an event file, with premonitory urges constituting the perceptual part and the motor or vocal expression constituting the action part coded in an event file [4]. Derived from this, GTS is assumed to be characterized by enhanced perception–action binding, i.e., “hyperbinding”. Several studies support the assumption that the TEC is a suitable framework to conceptualize GTS as it takes perception and action processes into account. A previous study investigating perception–action integration during a response selection (stimulus–response) task did indeed confirm the notion of “hyperbinding”, i.e., showed increased perception–action binding effects in adult patients with GTS compared to healthy controls [70]. Furthermore, tic frequency correlated with performance in conditions where unbinding processes of previously established perception–action bindings were required, with higher tic frequency being associated with stronger perception–action binding effects. Another study investigating stimulus–response binding during response inhibition revealed evidence of hyperbinding effects in children and adolescents with GTS [71]. The false alarm rate in a multi-modal Go/NoGo task was higher when response inhibition was triggered by unimodal (visual) input, demonstrating hyperbinding between bimodal input and the associated response, resulting in larger costs when a reconfiguration of the stimulus–response association was necessary in trials with unimodal input. The significant association between tic frequency and the degree of stimulus–response binding suggests that perception–action hyperbinding might be essential in understanding the mechanisms underlying GTS, therefore potentially making it relevant to treatment and in a clinical sense [70].

However, to date, it remains unclear whether the differences between patients with GTS and healthy control subjects can be attributed to altered binding, retrieval, or a combination of both. This further differentiation must be taken into account in future research to better understand altered mechanisms in GTS. For instance, behavioral strategies to normalize increased binding in GTS might be designed to either focus on binding or retrieval as subprocesses of perception–action integration depending on which of these subprocesses is predominantly altered in these patients. TEC and, more specifically, BRAC offers a new framework for investigating and explaining the mechanisms underlying the effects of established behavioral interventions such as CBIT/HRT and ERP.

## 7. Cognitive and Neural Mechanisms Underlying CBIT/HRT and ERP in Light of TEC and BRAC

Understanding neurophysiological mechanisms in the context of TEC and BRAC requires specific EEG analysis methods that emphasize the distinction between perceptual and motor related processes and perception–action integration, i.e., object, action, and event files.

Previous studies have shown that perception–action integration processes can be best examined when decomposing recorded EEG data [72,73,74]. This can be performed by using the residue iteration decomposition (RIDE) algorithm [75,76], which dissociates three clusters of activity: the S-cluster reflecting stimulus-related information, the R-cluster encompassing information related to motor processes, and an intermediate C-cluster located between S- and R-cluster reflecting response selection processes based on stimulus and response features [77]. The decomposed S-, C-, and R-clusters resemble the TEC concepts of the object, action, and event files, and neurophysiological studies have already provided evidence supporting the validity of this assumption [72,73]. The S-cluster is hypothesized to reflect object file processes, the R-cluster is hypothesized to reflect action file processes, and the C-cluster is assumed to depict stimulus–response bindings, i.e., event file processes. Based on this analogy, neurophysiological mechanisms can be explained using TEC as a framework.

A previous study [70] revealed that behavioral “hyperbinding” effects observed in adult patients with GTS were paralleled by amplitude modulations in the C-cluster (P3) time window reflecting stimulus–response translation processes, i.e., how specific stimulus features are related to a certain response [78]. Other studies also suggest the suitability of the C-cluster as a neurophysiological correlate of event file processes [72,73,74]. These results and the finding of absent alterations in action file processes in GTS [56] further support the conceptualization of GTS as a perception–action integration rather than a pure movement disorder [4,70]. On a behavioral level, hyperbinding during response selection was not observed in adolescents with GTS, yet the brain-oscillatory neuroanatomical basis of perception–action integration deviated from healthy controls [54]. On the other hand, hyperbinding was observed during inhibitory control in children and adolescents with GTS and was also associated with C-cluster modulations [71]. The concept of perception–action hyperbinding derived from TEC seems to be very relevant in understanding the mechanisms underlying GTS. In the future, the TEC/BRAC concept could be applied in order to improve neuropsychological assessments of GTS and other disorders. In addition, the correlation of clinical signs and hyperbinding further underscores its relevance for the deeper understanding of mechanistic principles of treatment effects. It can be hypothesized that behavior therapy effectively leads to tic disorder improvement through the reconfiguration of event file bindings.

## 8. CBIT/HRT and ERP Mechanisms of Action Conceptualized by TEC/BRAC

The conceptualization of urges (perception) and tics (action) as an event file allows for the investigation of working principles of CBIT and ERP in the context of TEC/BRAC. During CBIT/HRT, patients are trained to act differently (i.e., perform the competing response) in response to the identical sensory input (i.e., premonitory urge), which, in TEC terms, requires a restructuring of tic-specific event files. Thus, the next time the sensory input is perceived (the urge), the re-configured event-file is retrieved, including the tic-incompatible response; thus, CBIT/HRT changes the actions that are retrieved by urges. It has already been shown that hyperbinding during inhibitory control in children and adolescents with GTS was no longer present after CBIT [19]. However, the study was not sufficiently controlled to allow stringent mechanistic interpretation and did not include neurophysiological data.

The TEC/BRAC framework may also be suitable for the explanation of the mechanistic principles underlying ERP treatment effects since ERP aims to cancel stimulus–response couplings. Although ERP is based on the disruption of stimulus–response binding, i.e., the urge-tic coupling [30], the exact mechanisms of action are still unclear [79]. Patients learn to inhibit tics in response to a premonitory urge [30,33], i.e., instead of executing a tic, complete response inhibition is required, following the same sensory input (premonitory urge), so that the existing urge-tic association is de-coupled. In terms of TEC/BRAC, it can be assumed that ERP facilitates the unbinding of tic-specific event files by de-coupling urges (sensory input) and tics (motor output) through improved inhibitory control. It has been shown that NoGo-responses are bound together with stimuli-like “normal” responses—also found in applied settings [80]. Thus, trying to inhibit the tic will lead to binding between the urge and no movement; in the same vein as for CBIT/HRT, the next time the urge is perceived, the NoGo-response or inhibition is retrieved, which in turn should facilitate inhibitory control for the current urge. Thus, the original tic event file will eventually be unbound.

**Figure 2:**
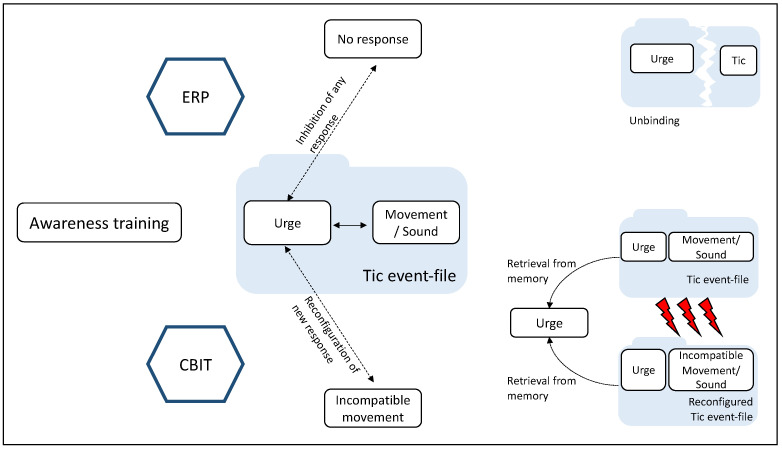
Illustration of the assumed mechanisms of the unbinding and reconfiguration of event files during CBIT and ERP according to TEC/BRAC. ERP targets the coupling of urges and tics and leads to unbinding by suppressing the tic response. CBIT predominately targets the retrieval of tic event files and reconfigured tic event files. CBIT leads to the retrieval of an alternative response, i.e., the urge causes memory retrieval of the tic response and the newly learned incompatible response and prevents tic execution once the new response is established.

Figure 2 illustrates the assumed mechanisms of action regarding CBIT/HRT and ERP against the conceptual framework of the TEC and BRAC.

Whereas, during CBIT, a new action is practiced in response to an identical sensory input, ERP teaches patients to perform no response to an unchanged sensory input. Therefore, CBIT may affect stimulus–response binding in the context of response selection when the urge retrieves competing event files since a competing response has to be executed to unchanged sensory input, whereas ERP effects presumably unfold during stimulus–response binding in the context of response inhibition as the intervention aims to increase inhibitory capacities and unbind the urge with the tic-specific movements. It is important to focus on the conceptual links between mechanisms underlying behavior therapy and TEC-related mechanisms, taking task performance in paradigms derived from TEC and associated neurophysiological markers into account. As mentioned earlier, a study has been conducted to classify the treatment effects of CBIT against the backdrop of TEC, although only the behavioral level was considered [19]. To date, no publication has addressed the question of the cognitive and neural mechanisms behind CBIT and ERP using TEC and BRAC. Based on behavioral and neurophysiological findings that revealed increased perception–action binding in patients with GTS during response selection [70] and inhibition [19,71] associated with C-cluster modulations, respectively, binding is likely to be increased prior to CBIT or ERP treatment and is associated with modulations in the C-cluster in the N2/P3 time window that reflect event file processes. After treatment, neurophysiological markers are assumed to no longer differ between patients and healthy controls. It is still unclear whether the mechanisms underlying CBIT and ERP are different or quite similar. It has to be clarified whether, in terms of TEC/BRAC, underlying mechanisms are based on an attenuation of event file hyperbinding by facilitating re-binding and, if so, whether it applies to both behavior therapies to the same extent. Since ERP focusses more on enhancing inhibitory processes, the modification of stimulus–response binding can probably be investigated particularly well during response inhibition. However, during CBIT, an alternative behavior other than the tic is trained, meaning that stimulus–response binding is presumably best investigated during response selection.

## 9. Conclusions and Outlook

HRT/CBIT and ERP are established and recommended methods in the treatment of tic disorders [11]. Particularly, the efficacy of HRT/CBIT has been proven in several studies, while less empirical evidence is available for ERP [11,38]. The exact mechanisms underlying the effectiveness of recommended behavioral interventions remain elusive. Habituation has been suggested as a mechanism underlying CBIT and ERP [20,30]. However, the habituation hypothesis is not undisputed [34]. We suggest another approach, conceptualizing tics as phenomena of perception–action hyperbinding based on the TEC and BRAC framework. Further research is needed to clarify whether binding or retrieval processes (or both) are altered in GTS and how recommended behavioral interventions affect these processes.

A few studies have already investigated the neurophysiological processes which change due to cognitive-behavioral therapy [49,53]. However, it has proven useful to consider GTS as a disorder of perception–action integration since there is ample evidence confirming the suitability of TEC and BRAC as a conceptual framework for GTS [19,54,55,57], resulting in new perspectives on the analysis of underlying mechanisms [4]. However, further investigations are needed to enhance the understanding of neurophysiological processes to enable the individualization of therapeutic approaches, i.e., the assignment of therapies according to predicted efficacy. Taking neurophysiological findings into account, this should be investigated in further studies to increase the rates of treatment response. Additionally, including underlying neurophysiological processes in the psychoeducational part of interventions could contribute to an increase in compliance and therapy motivation, which would eventually increase therapy satisfaction and efficacy.

Moreover, further behavioral therapy methods, including third-wave procedures such as metacognitive therapy [81] or acceptance and commitment therapy [82] in which mindfulness and attention training are significant components, should be considered in the treatment of GTS in order to increase the proportion of treatment responders, which is only about 50% with the psychotherapeutic approaches recommended to date [83]. During attention training, which is, among others, an important component of metacognitive therapy [83], attention is focused on external stimuli, leading to tic reduction, provided that the tics are not suppressed [84,85]. Initial pilot studies have been conducted to investigate the effectiveness of attention training [83] and acceptance and commitment therapy [86] in reducing tics; however, to date, there is insufficient evidence to recommend these methods in the treatment of tic disorders [83,86]. There is still a need to evaluate the effectiveness of attention- and mindfulness-based behavior therapy interventions in the treatment of tic disorders. Furthermore, factors such as comorbidities and other characteristics among patients should be examined, as they can influence which intervention is most likely to be effective. Again, neurophysiological markers should be examined to investigate the principles of action of different behavioral therapy approaches in order to individualize the allocation of therapy.

We conclude that expanding the selection of behavioral therapies in relation to the treatment of tic disorders and clarifying the underlying mechanisms are clinically highly relevant issues that need to be addressed in the future in order to foster the individualization and personalization of behavioral interventions.

## Data Availability

All relevant data are within the manuscript.
